# The Quantitative Associations Between Near Infrared Spectroscopic Cerebrovascular Metrics and Cerebral Blood Flow: A Scoping Review of the Human and Animal Literature

**DOI:** 10.3389/fphys.2022.934731

**Published:** 2022-07-15

**Authors:** Alwyn Gomez, Amanjyot Singh Sainbhi, Logan Froese, Carleen Batson, Trevor Slack, Kevin Y. Stein, Dean M. Cordingley, Francois Mathieu, Frederick A. Zeiler

**Affiliations:** ^1^ Department of Human Anatomy and Cell Science, Rady Faculty of Health Sciences, University of Manitoba, Winnipeg, MB, Canada; ^2^ Section of Neurosurgery, Department of Surgery, Rady Faculty of Health Sciences, University of Manitoba, Winnipeg, MB, Canada; ^3^ Biomedical Engineering, Faculty of Engineering, University of Manitoba, Winnipeg, MB, Canada; ^4^ Applied Health Sciences Program, University of Manitoba, Winnipeg, MB, Canada; ^5^ Pan Am Clinic Foundation, Winnipeg, MB, Canada; ^6^ Interdepartmental Division of Critical Care, Department of Medicine, University of Toronto, Toronto, ON, Canada; ^7^ Centre on Aging, University of Manitoba, Winnipeg, MB, Canada; ^8^ Division of Anaesthesia, Department of Medicine, Addenbrooke’s Hospital, University of Cambridge, Cambridge, MA, United Kingdom; ^9^ Department of Clinical Neuroscience, Karolinska Institutet, Stockholm, Sweden

**Keywords:** biomedical optics, cerebral blood flow (CBF), cerebrovascular physiology, near infrared spectroscopy, scoping review

## Abstract

Cerebral blood flow (CBF) is an important physiologic parameter that is vital for proper cerebral function and recovery. Current widely accepted methods of measuring CBF are cumbersome, invasive, or have poor spatial or temporal resolution. Near infrared spectroscopy (NIRS) based measures of cerebrovascular physiology may provide a means of non-invasively, topographically, and continuously measuring CBF. We performed a systematically conducted scoping review of the available literature examining the quantitative relationship between NIRS-based cerebrovascular metrics and CBF. We found that continuous-wave NIRS (CW-NIRS) was the most examined modality with dynamic contrast enhanced NIRS (DCE-NIRS) being the next most common. Fewer studies assessed diffuse correlation spectroscopy (DCS) and frequency resolved NIRS (FR-NIRS). We did not find studies examining the relationship between time-resolved NIRS (TR-NIRS) based metrics and CBF. Studies were most frequently conducted in humans and animal studies mostly utilized large animal models. The identified studies almost exclusively used a Pearson correlation analysis. Much of the literature supported a positive linear relationship between changes in CW-NIRS based metrics, particularly regional cerebral oxygen saturation (rSO_2_), and changes in CBF. Linear relationships were also identified between other NIRS based modalities and CBF, however, further validation is needed.

## Introduction

Cerebral blood flow (CBF), defined as the volume of blood flowing through a mass of brain over a period of time, is integral to brain function and a vital parameter in evaluating brain health (Fantini et al., 2016). Generally, the examination of CBF can be thought of either on the arterial scale or on the microvascular scale (arteries, capillaries, and venules). There are several existing methodologies that have been widely applied in the quantitative measurement of CBF on both scales. These included methods such as microsphere (MS) techniques that require sacrifice of the subject prior to measurement of CBF, making them only suitable for animal use. Alternatively, early non-invasive studies utilized a xenon^133^ clearance method (Thomas et al., 1979) while contemporary methods also exist such as positron emission tomography (PET) imaging (Frackowiak et al., 1980), perfusion weighted magnetic resonance imaging (PW-MRI; Zaro-Weber et al., 2009), arterial spin labelling MRI (ASL-MRI; Parkes et al., 2004), functional MRI derived CBF (fMRI-CBF; Chen et al., 2008), perfusion computed tomography (CTP; Hamberg et al., 1996), xenon-enhanced computed tomography (Xe-CT; Meyer et al., 1981), and single-photon emission computerized tomography (SPECT) imaging (Rootwelt et al., 1986). These methods, however, are generally cumbersome and have poor temporal resolution; some also involve the use of ionizing radiation. Temporal resolution can be improved by utilizing methods such as laser Doppler (Dirnagl et al., 1989) or thermal diffusion flowmetry (Mathieu et al., 2019). However, these methods only measure CBF in a small region of the brain and their invasive nature limits their scalability in the same subject.

Near infrared spectroscopy (NIRS) was first described as a means of evaluating cerebrovascular physiology in 1977 by Franz Jöbsis (Jöbsis, 1977). Fundamentally, this technology leverages the ability of near infrared (NIR) light (650–950 nm) to penetrate deep into tissues and be absorbed by a few physiologic chromophores including oxygenated and deoxygenated hemoglobin (OxHgB and deOxHgB respectively). Through the modified Beer-Lambert law the interaction of NIR light with tissues can be used to derive approximations of OxHgB and deOxHgB concentration (Cope, 1991). These in turn can be utilized to derive other parameters such as the total concentration of hemoglobin (tHgB) or the regional tissues oxygenation (rSO_2_) which is simply a function of the ratio of OxHgB to tHgB (Cope, 1991). The penetrative nature of NIRS allows it to act as a non-invasive continuous means of evaluating cerebrovascular physiology.

There are various modalities of NIRS that function on different variations of this principle. These included continuous wave NIRS (CW-NIRS), frequency-resolved NIRS (FR-NIRS), time-resolved NIRS (TR-NIRS), diffuse correlation spectroscopy (DCS), and dynamic contrast-enhanced NIRS (DCE-NIRS). CW-NIRS measures the degree of light at a fixed wavelength that is absorbed to determine a concentration of a particular chromophore. FR-NIRS modulates the intensity of its light source and measures the associated phase shift as it passes through the tissue. TR-NIRS generates ultrashort pulses of light and examines the temporal point spread function as the light emerges from the tissue. DCS examines the temporal fluctuations of reflected NIR light to produce a measure of CBF (CBF_DCS_). Finally, DCE-NIRS utilizes an optical tracer and applies the Fick principle to produce a measure of CBF (CBF_DCE-NIRS_). A detailed discussion of the technical nuances of these modalities is beyond the scope of this article. The interested reader is directed to comprehensive reviews of these technologies already present in the literature (Ferrari et al., 2004; Highton et al., 2010; Elwell and Cooper, 2011; Pellicer and Bravodel, 2011; Smith, 2011; Ghosh et al., 2012).

The non-invasive, continuous, and regional nature of NIRS makes it an ideal prospective method of evaluating cerebrovascular physiologic parameters as both the temporal evolution and topographic distribution of these parameters can be examined in a variety of settings and subjects. What is unclear is the association between these various parameters and CBF. Prior to NIRS becoming a “widely accepted” means of evaluating CBF the precise nature of this relationship must be understood. As such, the aim of this study was to provide a systematically conducted scoping review of the available human and animal literature that quantitatively examines the relationship between NIRS based cerebrovascular parameters and “widely accepted” measures of CBF.

## Methods

Utilizing the methodological framework described by Arksey and O’Malley (Arksey and O’Malley, 2005), a systematically conducted scoping review of the available English language literature was conducted. The Preferred Reporting Items for Systematic Reviews and Meta-Analyses Extension for Scoping Reviews (PRISMA-ScR) guidelines were followed (Tricco et al., 2018). The methodology, including the search strategy, is similar to other scoping reviews published by our group (Froese et al., 2020a, 2020b, 2020c; Gomez et al., 2021b). Given that this is a systematic scoping review of the literature it was not eligible for registration in PROSPERO.

The review question and search strategy were decided upon by the senior author (F.A.Z.) and primary author (A.G.).

### Search Questions, Populations, and Inclusion/Exclusion Criteria

The question for this systematically conducted scoping review is as follows: What is the quantitative relationship between NIRS-based cerebrovascular metrics and CBF in humans and animals?

All English language studies, in humans or animals, that quantitatively examined the relationship between any NIRS-based cerebrovascular metric and a “widely accepted” measure of CBF were included. Any modality of NIRS was included, so long as it was aimed at measuring a cerebrovascular parameter. This included CW-NIRS, FR-NIRS, TR-NIRS, and DCE-NIRS as well as DCS. A full description of these methodologies can be found in a previous review by this group (Gomez et al., 2021c). “Widely accepted” measures of CBF included the following: Xenon^133^ clearance, MS techniques (radiolabelled, florescent, or coloured), laser Doppler flowmetry, thermal diffusion flowmetry, PET imaging, PW-MRI, ASL-MRI, fMRI-CBF, CTP, Xe-CT, and SPECT. The primary outcome of interest was the quantifiable relationship between NIRS-based cerebrovascular metrics and these measures of CBF.

Studies were excluded if there was no “widely accepted” measure of CBF. Notable methods that were not considered “widely accepted” measures of CBF included transcranial Doppler ultrasonography, as this method is reliant on a constant vessel diameter to be linearly related to CBF over time (Gomez et al., 2021a), and blood-oxygen-level-dependent functional magnetic resonance imaging (BOLD-fMRI), which only measures changes in oxygenated blood flow (Hillman, 2014). Additionally, studies that did not have quantitative measures of the relationship between NIRS-based cerebrovascular metrics and CBF were also excluded. Therefore, studies that only presented qualitative trends were not included. While significant advancements have been made in recent years with regards to computational models and simulations of CBF (Lan et al., 2020; Xie et al., 2022), for the purposes of this review *in silico* studies examining the relationship between NIRS based metrics and CBF were also excluded. Finally, review articles and non-English language articles were also excluded from consideration.

### Search Strategy

BIOSIS, Cochrane Library, EMBASE, MEDLINE, and SCOPUS were searched from the inception of each database to February 2022 using tailored search strategies for each database. As an example, the search strategy for BIOSIS, including the keywords utilized, can be seen in Supplementary Appendix A1. Similar strategies were used for each of the other databases. Search results were then combined, and deduplication was performed.

### Study Selections

Using two reviewers (AG and AS), a two-step manual review of all articles returned by the search strategy was performed. In the first filter phase, each reviewer independently screened all studies identified using the above-described search strategy and determined if they met the inclusion criteria based on their title and abstract. The resulting list of studies was then passed through the second filter phase where once again each reviewer independently determined if the studies met the inclusion criteria, but this time based on the full text. Any discrepancies between the two reviewers were resolved by a third party (FZ). For any conference abstracts that were identified during this process an effort was made to identify associated peer-reviewed manuscripts for inclusion. Finally, the reference section of each article included in the final review were also examined to ensure no studies were missed.

### Data Collection

Data was extracted from the final list of articles and compiled into various data fields. These fields included: the species of the subject examined and their clinical condition, the number of subjects, the experimental conditions, the “widely accepted” measure of CBF utilized, the NIRS modality or modalities utilized, the NIRS device utilized, the NIRS parameters examined, the results of the analysis of association, and any study limitations.

### Bias Assessment

Given the goal of this review was to provide a comprehensive scoping overview of the available literature, a formal bias assessment was not conducted.

### Statistical Analysis

Due to the heterogeneity of the results/study designs and that the goal of the study was to conduct a scoping overview, no meta-analysis was performed.

## Results

### Search Results and Study Characteristics

**FIGURE 1:**
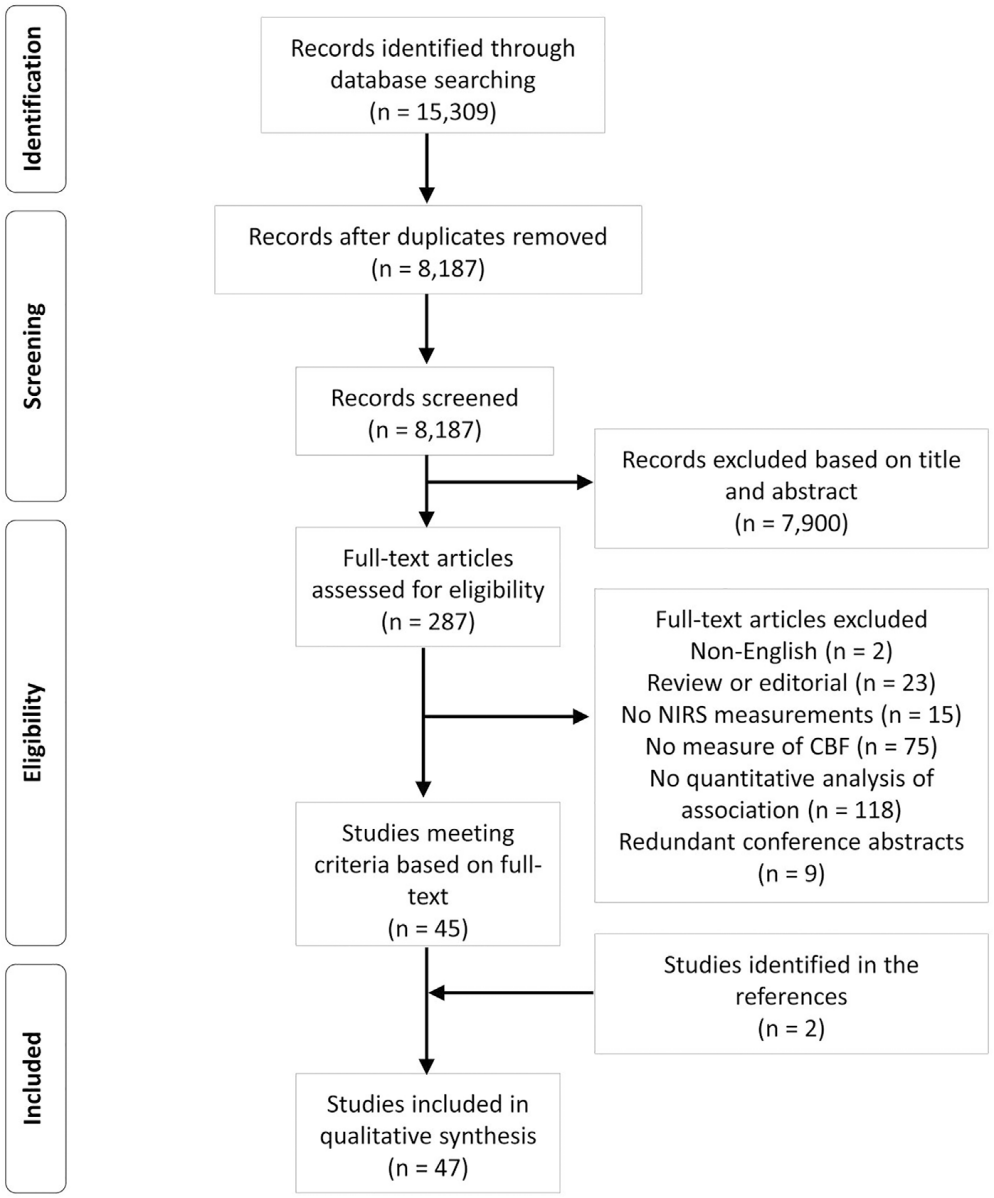
A PRISMA flow-diagram of the systematically conducted scoping review of the literature.

The overall search and filtration results have been summarized in Figure 1 using a PRISMA flow-diagram. The combined results from the search, carried out over all five databases, yielded 15,309 articles. Following deduplication 8,187 unique articles were identified. During the first filter phase, 7,900 articles were found to not meet the inclusion/exclusion criteria based on their title and abstract. This left 287 articles for which the full text was reviewed in the second filter phase. After reviewing the full text, 242 articles were found to not meet inclusion/exclusion criteria leaving 45 articles. Examination of the reference sections of those texts yielded two additional articles resulting in 47 studies being included in this scoping review.

A number of different NIRS modalities were examined for their association with CBF. CW-NIRS was the most frequently utilized modality and was compared to CBF in 28 studies (Pryds et al., 1990, 2005; Skov et al., 1991; Bucher et al., 1993; Kaminogo et al., 1995; Kirkpatrick et al., 1995; Hock et al., 1997; Holzschuh et al., 1997; Newton et al., 1997; Villringer et al., 1997; Goddard-Finegold et al., 1998; Pellicer et al., 1999; Yamaguchi et al., 1999; Soul et al., 2000; Ogasawara et al., 2003; Huppert et al., 2006; Murata et al., 2006; Ricci et al., 2006; Naulaers et al., 2007; Themelis et al., 2007; Kim et al., 2010; Taussky et al., 2012; Alderliesten et al., 2014, 2017; Wintermark et al., 2014; Alosh et al., 2016; Polinder-Bos et al., 2020; Hashem et al., 2022). DCE-NIRS was the next most commonly used modality with its relationship to CBF examined in 12 studies (Kuebler et al., 1998; Roberts et al., 1998; Brown et al., 2001, 2002; De Visscher et al., 2003; Keller et al., 2003; Rothoerl et al., 2003; Schytz et al., 2009; Elliott et al., 2010, 2014; Saito et al., 2018; Milej et al., 2020). Of note, while most DCE-NIRS studies utilized continuous-wave technology (Kuebler et al., 1998; Roberts et al., 1998; Brown et al., 2001, 2002; De Visscher et al., 2003; Keller et al., 2003; Rothoerl et al., 2003; Schytz et al., 2009; Saito et al., 2018), two studies examined contrast signals utilizing time-resolved optical analysis (Elliott et al., 2014; Milej et al., 2020). DCS was utilized to measure CBF in seven studies (Zhou et al., 2009; Carp et al., 2010; Durduran et al., 2010; Kim et al., 2010; Shang et al., 2011; Baker et al., 2019; Giovannella et al., 2020), however five of these studies combined DCS with either FR-NIRS (Carp et al., 2010), CW-NIRS (Zhou et al., 2009; Durduran et al., 2010), or TR-NIRS (Baker et al., 2019; Giovannella et al., 2020) into a hybrid device for the purposes of improving the estimation of CBF. There was only one study that measured cerebrovascular parameters using FR-NIRS(Kurth et al., 2002) and no studies were found that compared TR-NIRS parameters and CBF directly. Table 1 Table, 2 to Table 3 summarize the findings of the various CW-NIRS, DCE-NIRS, and DCS studies respectively.

**TABLE 1:** Summary of articles examining continuous wave near infrared spectroscopic (CW-NIRS) cerebrovascular parameters.

Article	Study subjects	Experimental conditions	Number of subjects	Measure of CBF	NIRS device	NIRS parameter examined	Study results and conclusions	Study limitations
**Human studies**								
Pryds et al. (1990)	Human neonates	• Mechanically ventilated preterm infants	24	Xe^133^ clearance	Research device	CBV_I_ (a function of tHgB)	• Strong linear Pearson correlation between the change in CBV_I_ and change in global CBF (R = 0.70; *p* < 0.0001)	• Change in CBF only induced through cerebral vasodilation. Limits the generalizability of this result
		• Measurements taken at various ETCO_2_ levels					• Slope of this relationship was 0.63 (95% CI: 0.40–0.87)	• CBF measured globally and not only at the region of interest interrogated by NIRS.
								• Only relative changes in the parameters from baseline were examined
								• No bias assessment performed
Skov et al. (1991)	Human neonates	• Mechanically ventilated preterm infants	16	Xe^133^ clearance	Research device	CBF_NIRS_ (A function of OxHgB and deOxHgB; utilizing the Fick principle with oxygen as a tracer)	• Strong linear Pearson correlation between absolute CBF_NIRS_ and global CBF (R = 0.917; *p* < 0.0001)	• CBF measured globally and not only at the region of interest interrogated by NIRS.
		• Measurements were taken at rest					• Slope of the relationship was 0.75 (SE = 0.064) with an intercept of 1.58	• Effect of changes in oxygen level on cerebral vasculature unaccounted for
		• Step changes in oxygen with it used as an endogenous tracer					• By Bland-Altman analysis, a trend for CBF_NIRS_ to underestimate CBF at higher values observed	
Bucher et al. (1993)	Human neonates	• Mechanically ventilated preterm infants	9	Xe^133^ clearance	Hamamatsu NIR-1000	CBF_NIRS_ (A function of OxHgB and deOxHgB; utilizing the Fick principle with oxygen as a tracer)	• Strong linear Pearson correlation between absolute CBF_NIRS_ and global CBF (R = 0.80; *p* < 0.001)	• CBF was measured globally and not only at region of interest interrogated by NIRS.
		• Measurements were taken at rest					• Slope of the relationship was 1.83 (SD 0.43) with an intercept of −8.8 (SD 5.46)	• Effect of changes in oxygen level on cerebral vasculature unaccounted for
		• Step changes in oxygen with it used as an endogenous tracer					• By Bland-Altman analysis, the average difference between the methods was 1.6 ml/100 g/min (95% LoA: 0.54–3.78 ml/100 g/min)	
							• Trend for CBF_NIRS_ to overestimate CBF at higher values	
Kaminogo et al. (1995)	Adult cerebral ischemia patients and healthy human controls	• Group of 7 cerebral ischemia patients and 5 healthy human controls	12	Xe^133^ clearance	INVOS-3100	rSO_2_ (a ratio of OxHgB to tHgB)	• Moderate linear Pearson correlation between change in rSO_2_ and percent change in rCBF (R = 0.521; *p* < 0.01)	• Change in CBF only induced through cerebral vasodilation. Limits generalizability of this result
		• Measurements taken during step changes in CBF induced by acetazolamide					• Slope of the relationship was 0.07 with an intercept of 2.15	• CBF was measured globally and not only at region of interest interrogated by NIRS.
								• Only relative changes in parameters from baseline were examined
								• No bias assessment performed
Kirkpatrick et al. (1995)	Adult severe TBI patient	• Single TBI patient	1	Laser Doppler flowmetry	Hamamatsu NIR-1000	OxHgB, deOxHgB, and tHgB	• Strong linear Pearson correlation between OxHgB and laser Doppler flowmetry (R = 0.71; no *p*-value reported)	• Relationship was only evaluated in one patient over a very short period
		• Measurements made during a 3-min period of elevated ICP						
Hock et al. (1997)	Alzheimer disease patients	• Group of Alzheimer disease patients	10	PET imaging	Hamamatsu NIR-500	OxHgB, deOxHgB, and tHgB	• Very strong linear Pearson correlation between change in tHgB and percent change in rCBF; was maximal at a depth of 0.675 cm (R = 0.925; *p* = 0.000)	• No statistical correction made for multiple comparisons
		• Measurements were made during performance of the Stroop test					• Slope of the relationship was 0.02077 with an intercept of -0.1443	• Only relative changes in parameters from their baseline were examined
							• Strong linear Pearson correlation between change in OxHgB and percent change in rCBF; was maximal at a depth of 0.675 cm (R = 0.754; *p* = 0.012)	• No bias assessment performed
							• Negative linear Pearson correlation between change in deOxHgB and percent change in rCBF that was maximal at a depth of 0.90 cm (R = -0.6852; *p* = 0.029)	
							• Correlations between changes in rCBF and NIRS parameters were best between a depth of 0.45 and 2.7 cm	
Holzschuh et al. (1997)	Adult cerebral ischemia patients	• Group of cerebral ischemia patients	21	Xe^133^ clearance	INVOS 3100	rSO_2_ (a ratio of OxHgB to tHgB)	• No linear correlation between absolute rSO_2_ and CBF	• Change in CBF was only induced through cerebral vasodilation. Limits the generalizability of this result
		• Measurements made during step changes in CBF induced by acetazolamide					• Strong linear Pearson correlation between change in rSO_2_ and change in CBF (R = 0.71, *p* < 0.05).	• CBF measured globally and not only at the region of interest interrogated by NIRS.
								• Only relative changes in parameters from baseline were examined
								• No bias assessment performed
Villringer et al. (1997)	Healthy adult humans	• Group of healthy adults	5	PET imaging	Hamamatsu NIRO 500	OxHgB, deOxHgB, and tHgB	• Very strong linear Pearson correlation between change in tHgB and change in rCBF; maximal at a depth of 0.90 cm (R = 0.88; *p* = 0.048)	• No statistical correction made for multiple comparisons
		• Measurements made during performance of the Stroop test					• No significant correlation between change in OxHgB or change in deOxHgB and change in rCBF.	• Only relative changes in parameters from baseline were examined
								• No bias assessment performed
Yamaguchi et al. (1999)	Adult cardiac surgery patients	• Group of adult cardiac surgery patients	10	SPECT	SHIMADZU OM-200	OxHgB and rSO_2_ (a ratio of OxHgB to tHgB)	• No linear correlation between absolute rSO_2_ and CBF.	• Change in CBF was only induced through cerebral vasodilation, limits generalizability of this result
		• Measurements made during step changes in CBF induced by acetazolamide					• Strong linear Pearson correlation between percent change in OxHgB and percent change in rCBF (R = 0.758, *p* < 0.011)	• Only relative changes in parameters from their baseline were examined
							• Strong linear Pearson correlation between percent change in rSO_2_ and percent change in rCBF (R = 0.740, *p* < 0.014)	• No bias assessment performed
Ogasawara et al. (2003)	Adult cerebral ischemia patients	• Group of patients undergoing carotid endarterectomy	50	SPECT	Tosetec TOS-96	rSO_2_ (a ratio of OxHgB to tHgB)	• Fair linear Pearson correlation between percent change in rSO_2_ after declamping of the ICA and change in rCBF after surgery (R = 0.247 *p* = 0.0002)	• Measurements between modalities were not taken simultaneously
		• Measurements taken with NIRS intraoperatively (before ICA clamping, after ICA declamping, and at end of the procedure); compared to pre- and post-operative SPECT studies					• Slope of the relationship was 4.4 with an intercept of 11.6	• Only relative changes in parameters from their baseline were examined
							• Strong linear Pearson correlation between percent change in rSO_2_ at end of procedure and change in rCBF after surgery (R = 0.822 *p* < 0.0001)	• No bias assessment performed
							• Slope of the relationship was 5.5 with an intercept of 7.5	
Huppert et al. (2006)	Healthy adult humans	• Group of healthy adult humans	5	ASL-MRI	Research device	OxHgB, deOxHgB, and tHgB	• Strong linear Pearson correlation between change in OxHgB and change in rCBF (R = 0.83, *p* < 0.001)	• Significant drop out in the initial cohort of 11 subjects due to technical problems
		• Measurements were taken during a finger-tapping task					• Strong linear Pearson correlation between change in tHgB and change in rCBF (R = 0.91, *p* < 0.001)	• No bias assessment performed
							• No significant linear correlation between change in deOxHgB and change in rCBF.	
Murata et al. (2006)	Adult cerebral ischemia patients and healthy adult human controls	• Group of adults with cerebral ischemia	20	SPECT	Hamamatsu NIRO 300	deOxHgB, and tHgB	• Significant positive linear Pearson correlation between tHgB and rCBF (*p* < 0.01; no R value reported)	• Correlation coefficients were not reported
		• Measurements were taken at rest					• Significant negative linear Pearson correlation between deOxHgB and rCBF (*p* < 0.01; no R value reported)	• No bias assessment performed
Kim et al. (2010)	Adult brain injury patients	• Group of critically ill adult brain injury patients	7	Xe-CT	Research device	OxHgB, deOxHgB, and tHgB	• Moderate non-significant linear Pearson correlation between change in OxHgB and rCBF (R = 0.57; *p* = 0.053)	• Only relative changes in parameters from their baseline were examined
		• Measurements were taken during various interventions to modify CBF; included manipulating ABP and ETCO_2_ levels					• No linear correlation between change in deOxHgB or tHgB and rCBF.	• No bias assessment performed
								• Effect of xenon inhalation on CBF potential confounder
Taussky et al. (2012)	Adult brain injury patients	• Group of critically ill adult brain injury patients	8	CTP	CAS Medical Systems FORE-SIGHT Cerebral Oximeter	rSO_2_ (a ratio of OxHgB to tHgB)	• Linear Pearson correlation between absolute rSO_2_ and rCBF (*p* < 0.0001; no R value given)	• Correlation coefficients not reported
		• Measurements were taken at rest						• No bias assessment performed
Alderliesten et al. (2014)	Healthy adult humans	• Group of healthy adult humans	7	fMRI (BOLD derived CBF)	Oxymon Mk III	rSO_2_ (a ratio of OxHgB to tHgB)	• Strong linear Pearson correlation between absolute rSO_2_ and rCBF (R = 0.85; *p* < 0.0001)	• Change in CBF only induced through cerebral vasodilation; limits generalizability of result
		• Measurements taken during modulation of ETCO_2_ levels						• No bias assessment performed
Wintermark et al. (2014)	Human newborns with hypoxic ischemic encephalopathy	• Group of newborns with hypoxic ischemic encephalopathy	7	ASL-MRI	CAS Medical Systems FORE-SIGHT Cerebral Oximeter	rSO_2_ (a ratio of OxHgB to tHgB)	• No Pearson correlation found between rSO_2_ and CBF over the entire cohort	• Measurements between modalities not taken simultaneously
		• Measurements taken during hypothermia					• Strong linear Pearson correlation between rSO_2_ and rCBF (R = 0.88; *p* < 0.01) in those with severe encephalopathy	• No bias assessment performed
Alderliesten et al. (2017)	Human neonates	• Group of critically ill neonates	15	ASL-MRI	Covidien INVOS 5100c	rSO_2_ (a ratio of OxHgB to tHgB)	• Strong linear Pearson correlation between absolute rSO_2_ and whole brain CBF (R = 0.71; *p* < 0.01)	• Measurements between modalities not taken simultaneously
		• Measurements taken at rest					• Slope of this relationship was 0.73 with an intercept of -38.8	• No bias assessment performed
							• Strong linear Pearson correlation between absolute rSO_2_ and rCBF (R = 0.844; *p* < 0.01)	
							• Slope of this relationship was 0.55 with an intercept of -30.2	
Polinder-Bos et al. (2020)	Adult end-stage renal disease patients	• Group of end-stage renal disease patients	12	PET imaging	Covidien INVOS 5100c	rSO_2_ (a ratio of OxHgB to tHgB)	• No Pearson correlation between the change in rSO_2_ and change in rCBF in left frontal gray matter	• Change in CBF only induced through volume depletion; limits generalizability of results
		• Measurements taken before, at start, and at end of dialysis					• Moderate Pearson correlation between change in rSO_2_ and rCBF in right frontal gray matter (R = 0.69; *p* = 0.03)	• No explanation was given for the hemispheric difference in results
							• Bland-Altman analysis showed a proportional bias; change in rCBF was underestimated by a change in rSO_2_ for larger rCBF values	
**Animal studies**								
Newton et al. (1997)	Mature dogs	• Group of healthy mechanically ventilated dogs	6	Radiolabeled microspheres	Hamamatsu NIRO 500	CBF_NIRS_ (A function of OxHgB and deOxHgB; utilizing the Fick principle with oxygen as a tracer)	• Moderate linear Pearson correlation between absolute CBF_NIRS_ and hemispheric CBF (R = 0.64 [0.63–1.00]; no *p*-value reported)	Change in CBF was only induced through cerebral vasodilation; limits generalizability of result
		• Measurements taken at various ETCO_2_ levels					• Strong linear Pearson correlation between absolute CBF_NIRS_ and gray matter CBF in region examined by NIRS (R = 0.77 [0.70–0.99]; no *p*-value reported)	• Effect of changes in oxygen level on cerebral vasculature unaccounted for
		• Step changes in oxygen allowed for it to be used as endogenous tracer					• By Bland-Altman analysis a trend for CBF_NIRS_ to underestimate CBF at higher values observed	• NIRS probes placed directly on the skull; limits generalizability to clinical applications
Goddard-Finegold et al. (1998)	Newborn piglets	• Porcine model of cerebral ischemia and reperfusion	13	Radiolabeled microspheres	Research device	CBF_NIRS_ (A function of OxHgB and deOxHgB; utilizing the Fick principle with oxygen as a tracer)	• Strong linear Pearson correlation between CBF_NIRS_ and CBF (R = 0.7096; *p* < 0.0001)	• CBF was measured globally and not only at region of interest interrogated by NIRS
		• Measurements were made using step changes in oxygen; allowed for it to be used as endogenous tracer					• Slope of the relationship was 0.747 with an intercept of 2.33	• Effect of changes in oxygen level on cerebral vasculature unaccounted.
							• By Bland-Altman analysis a trend for CBF_NIRS_ to overestimate CBF at higher values observed	
Pellicer et al. (1999)	Newborn piglets	• Group of healthy newborn piglets	15	Radiolabeled microspheres	CRITIKON Cerebral RedOx Monitor	CBV_NIRS_ (a function of tHgB)	• In individual subjects there was no agreement found between change in CBV_NIRS_ and change in CBF.	• No analysis performed over entire cohort
		• 7 administered ibuprofen and 8 administered saline						• CBF measured globally and not only at region of interest interrogated by NIRS.
		• Measurements were taken during hypercapnia induced vasodilation						• Only relative changes in the parameters from their baseline examined
Soul et al. (2000)	Newborn piglets	• Porcine model of hydrocephalus	7	Radiolabeled microspheres	Hamamatsu (specific device not specified)	ΔHgB (the difference between OxHgB and deOxHgB)	• There was a linear Pearson correlation between change in ΔHgB and change in rCBF in the cortex, white matter, and basal ganglia (*p* < 0.0001 in all; no R value given)	• Change in CBF was only induced through increases in ICP; limits generalizability of result
		• Measurements were taken during elevations in ICP induced by intraventricular infusion of fluid						• Correlation coefficients not reported
								• No bias assessment performed
								• Only relative changes in parameters from their baseline were examined
Pryds et al. (2005)	Rat pups	• Group of healthy rat pups	20	Radiolabeled microspheres	Research device	tHgB and ΔHgB (the difference between OxHgB and deOxHgB)	• Strong linear Pearson correlation between change in tHgB and percent change in rCBF (R = 0.86, *p* < 0.001)	• Only relative changes in parameters from baseline examined
		• Measurements taken during hemorrhage induced hypotension and hypercapnia induced vasodilation					• Strong linear Pearson correlation between change in ΔHgB and percent change in rCBF (R = 0.80, *p* < 0.001)	• No bias assessment performed
Ricci et al. (2006)	Newborn piglets	• Porcine model of aortopulmonary shunting	8	Radiolabeled microspheres	Somanetics INVOS 5100	rSO_2_ (a ratio of OxHgB to tHgB)	• Fair negative linear Pearson correlation between absolute rSO_2_ and CBF (R = -0.39; *p* = 0.05)	• CBF was measured globally and not only at region of interest interrogated by NIRS.
		• Measurements were taken at baseline					• Slope of this relationship was -0.19 with an intercept of 45.9	• No bias assessment performed
								• Validity of microsphere based CBF unclear in cardiac shunting model
Naulaers et al. (2007)	Newborn piglets	• Group of healthy newborn piglets	6	Coloured microspheres	Hamamatsu NIRO 300	TOI (a ratio of OxHgB to tHgB)	• No significant correlation found between TOI and CBF.	• CBF was measured globally and not only at region of interest interrogated by NIRS.
		• Measurements were taken at 33 , 35, 37 °C, and during hypocapnia						• No bias assessment performed
Themelis et al. (2007)	Newborn piglets	• Group of healthy newborn piglets	1	Laser Doppler flowmetry	TechEn Inc. NIRS 2	pCBF (a function of the peak rate of change in absorbance during each heartbeat)	• Strong linear Pearson correlation between pCBF and rCBF as measured by laser Doppler flowmetry (R = 0.978, no *p* value reported)	• Correlation analysis was only performed with data from one out of the 8 subjects
		• Measurements were taken during hypercapnia induced vasodilation						• No bias assessment performed
Alosh et al. (2016)	Newborn piglets	• Model of intracranial hypertension	7	Coloured microspheres	Intergra Life Sciences INVOS	rSO_2_ (a ratio of OxHgB to tHgB)	• Using power regression analysis, strong Pearson correlation was found between absolute rSO_2_ and rCBF (R = 0.95; *p* < 0.05)	• Change in CBF was only induced through increases in ICP; Limits generalizability of result
		• Measurements were taken during elevations in ICP induced by intraventricular infusion of fluid						• No bias assessment performed
								• No physiologic justification given for the use of power regression analysis over linear regression analysis
								• NIRS probes placed directly on the skull; limits generalizability to clinical applications
Hashem et al. (2022)	Mature mice	• Mouse model of demyelination and a group of healthy controls	20	ASL-MRI	Research device	rSO_2_ (a ratio of OxHgB to tHgB) and tHgB	• In control mice, no correlation was found between rSO_2_ or tHgB and rCBF	• Measurements between modalities not taken simultaneously
		• Measurements taken during hyperoxia, normoxia, and anoxia					• In demyelinated mice a strong negative Pearson correlation was found between tHgB and rCBF (R = -0.7; *p* = 0.02); no correlation was found between rSO_2_ and rCBF..	• During chromophore quantification OxHgB and tHgB not measured directly, only deOxHgB was. Parameters were derived based on assumption that tHgB = deOxHgB during anoxic period
								• No bias assessment performed

ASL-MRI, arterial spin labeling magnetic resonance imaging; CBF, cerebral blood flow; CBF_NIRS_, cerebral blood flow derived with near infrared spectroscopy; CTP, perfusion computed tomography; CVB_I_, cerebral blood volume index; CVB_NIRS_, cerebral blood volume derived with near infrared spectroscopy; ΔHgB = difference between oxygenated and deoxygenated hemoglobin; deOxHgB = deoxygenated hemoglobin; ETCO_2_ = end-tidal carbon dioxide; fMRI, functional magnetic resonance imaging; ICA, internal carotid artery; ICP, intracranial pressure; NIRS, near infrared spectroscopy; OxHgB = oxygenated hemoglobin; pCBF, pulsatile cerebral blood flow; PET, positron emission tomography; rCBF, regional cerebral blood flow; rSO_2_ = regional cerebral oxygen saturation; SPECT, single-photon emission computerized tomography; tHgB = total hemoglobin; TOI, tissue oxygen index; Xe = xenon; Xe-CT, xenon-enhanced computed tomography.

**TABLE 2:** Summary of articles examining dynamic contrast enhanced near infrared spectroscopic (DCE-NIRS) cerebrovascular parameters.

Article	Study subjects	Experimental conditions	Number of subjects	Measure of CBF	NIRS device	Contrast agent	Study results and conclusions	Study limitations
**Human studies**								
Keller et al. (2003)	Healthy adult humans	• Group of healthy adult humans	6	PW-MRI	Research device	ICG	• Strong linear correlation between CBF_DCE-NIRS_ and rCBF based on visual inspection; no regression analysis performed	• Measurements between modalities not taken simultaneously
		• Measurements taken with and without CPAP.					• By Bland-Altman analysis difference between modalities did not varied in any systemic way	• Regression analysis was not reported; reporting of bias assessment was incomplete
							• Large 95% limit of agreement at ± 76 ml/100 g/min	
Rothoerl et al. (2003)	Adult brain injury patients	• Group of 9 critically ill TBI patients and one critically ill SAH patient	10	Xe^133^ clearance	Somanetics INVOS 4100	ICG	• No correlation between CBF_DCE-NIRS_ and CBF regardless of method used	• rSO_2_ was used to monitor the transit of contrast bolus
		• Measurements were taken at rest						
Schytz et al. (2009)	Healthy adult humans	• Group of healthy adult humans	10	SPECT	TechEn Inc. NIRS 2	ICG	• No significant correlation was found between CBF_DCE-NIRS_ and CBF.	• CBF_DCE-NIRS_ unable to detect any change following administration of acetazolamide; calls to question the methodology utilized
		• Measurements were taken during changes in CBF induced by acetazolamide						
Saito et al. (2018)	Adult cerebral ischemia patients	• Group of adult cerebral ischemia patients	29	PET imaging	Hamamatsu NIRO-200 NX	ICG	• Moderate linear Pearson correlation in ratio of CBF_DCE-NIRS_ between hemispheres and ratio of rCBF between hemispheres (R = 0.618; *p* = 0.0004)	• Measurements between modalities not taken simultaneously
		• Measurements taken at rest					• Bland-Altman analysis showed minimal bias of -0.02 without trend; 95% limit of agreement ± 0.28	• Only hemispheric ratios examined and not absolute values of CBF.
Milej et al. (2020)	Healthy adult humans	• Group of healthy adult humans	10	ASL-MRI	Research device (Utilizing TR-NIRS)	ICG	• Strong linear Pearson correlation between CBF_DCE-NIRS_ and rCBF (R = 0.94; no *p*-value reported)	• Change in CBF was only induced through cerebral vasodilation; limits generalizability
		• Measurements were taken during hypercapnia induced vasodilation					• Slope of this relationship was 0.99 with an intercept of −1.7	• Measurements between modalities not taken simultaneously
							• Bland-Altman analysis showed minimal bias (1.9 ml/100 g/min) without trend; 95% limit of agreement ± 17 ml/100 g/min	• Methodology used required measurement of extracerebral tissue thickness by MRI prior to measurement of cerebrovascular parameters
**Animal studies**								
Kuebler et al. (1998)	Mature pigs	• Porcine models of coronary stenosis and hemorrhagic shock as well as in a group of healthy control	8	Radiolabeled microspheres	Hamamatsu NIRO 500	ICG	• Strong linear Pearson correlation between CBF_DCE-NIRS_ and cortical rCBF (R = 0.814; *p* < 0.001)	• Large limits of agreement likely due to modest sample size
		• Measurements were made at multiple timepoints					• Slope of this relationship was 1.004 with an intercept of -12.85	
							• No significant linear correlation between CBF_DCE-NIRS_ and galeal blood flow	
							• As depth increased the Pearson correlation between CBF_DCE-NIRS_ and CBF decreased with R = 0.793, 0.771, and 0.724 for white matter, basal brain regions, and basal ganglia respectively (*p* < 0.001 for all)	
							• Bland-Altman analysis showed minimal bias (0 ± 3.7 ml/100 g/min) without trend; 95% limits of agreement large at ± 38 ml/100 g/min	
Roberts et al. (1998)	Newborn piglets	• Group of healthy pigs	3	Radiolabeled microspheres	Hamamatsu NIRO 500	ICG	• Strong linear Pearson correlation between CBF_DCE-NIRS_ and rCBF within animals (R = 0.95 to 0.99; no *p*-value given)	• Subjects on cardiopulmonary bypass for the entirety of the measurements; limits generalizability
		• Measurements made on cardiopulmonary bypass					• Slope of this relationship ranged from 0.5 to 1.8	• Regression analysis only performed within individual subjects and not over cohort
								• Incomplete reporting of regression analysis and bias assessment
Brown et al. (2001)	Newborn piglets	• Group of healthy newborn piglets	3	CTP	Research device	ICG	• Strong linear Pearson correlation between CBF_DCE-NIRS_ and rCBF (R = 0.98; no *p*-value given)	• Change in CBF only induced through cerebral vasodilation; limits generalizability
		• Measurements were taken at various ETCO_2_ levels					• Slope of relationship was 1.05 with an intercept of -4.30	• No bias assessment performed
Brown et al. (2002)	Newborn piglets	• Group of healthy newborn piglets	6	CTP	Research device	ICG	• Bland-Altman analysis showed minimal bias (-2.05 ml/100 g/min) without trend; 95% limit of agreement ± 12.44 ml/100 g/min	• Change in CBF was only induced through cerebral vasodilation; limits generalizability
		• Measurements were taken at various ETCO_2_ levels						• Regression analysis was not reported
De Visscher et al. (2003)	Mature rats	• Group of healthy rats	36	Coloured microspheres	Research device	ICG	• Moderate linear Pearson correlation between CBF_DCE-NIRS_ and whole brain CBF (R = 0.6466; *p* < 0.0001)	• Proportion of the subjects had pharmacologically induced vasoconstriction; limits generalizability
		• Measurements taken in 12 at rest, 12 during 1-NAME induced, and 12 during hypercapnia induced vasodilation					• Slope of this relationship was 7.06 with intercept of 62.4	• Bias assessment was not fully reported
							• Bland-Altman analysis showed minimal bias not significantly different than zero; limits of agreement were large (approximately 100 ml/100 g/min based on plot)	• CBF was measured globally and not only at region of interest interrogated by NIRS.
Elliott et al. (2010)	Immature pigs	• Group of 1– to 2-month-old pigs	8	CTP	Research device	ICG	• Strong linear Pearson correlation between CBF_DCE-NIRS_ and rCBF (R = 0.845; *p* < 0.001)	• Change in CBF was only induced through cerebral vasodilation; limits generalizability
		• Measurements were taken at various ETCO_2_ levels					• Slope of this relationship was 0.92 with an intercept of 2.7	• Required measurement of extracerebral tissue thickness by CT
							• Bland-Altman analysis showed minimal bias (-2.83 ml/100 g/min) without trend; 95% limit of agreement was ± 16.8 ml/100 g/min	.
							• No correlation identified for non-depth resolved NIRS methods	
Elliott et al. (2014)	Mature pigs	• Group of healthy mature pigs	8	CTP	Research device (Utilizing TR-NIRS)	ICG	• Strong linear Pearson correlation between CBF_DCE-NIRS_ and rCBF (R = 0.86; no *p*-value reported)	• Measurements between modalities not taken simultaneously
		• Measurements were taken at baseline, during hypocapnia, and during ischemia					• Slope of this relationship was 1.06 with an intercept of -4.37	• Model of ischemia may not have uniformly affected region of interest
							• Bland-Altman analysis showed minimal bias (-1.7 ml/100 g/min) without trend; 95% limit of agreement ± 14.6 ml/100 g/min	

ASL-MRI, arterial spin labeling magnetic resonance imaging; CBF, cerebral blood flow; CBF_DCE-NIRS_, cerebral blood flow derived with dynamic contrast enhanced near infrared spectroscopy; CPAP, continuous positive airway pressure; CTP, perfusion computed tomography; deOxHgB = deoxygenated hemoglobin; ICG, indocyanine green; MRI, magnetic resonance imaging; NIRS, near infrared spectroscopy; OxHgB = oxygenated hemoglobin; PET, positron emission tomography; PW-MRI, perfusion-weighted magnetic resonance imaging; rCBF, regional cerebral blood flow; rSO_2_ = regional cerebral oxygen saturation; SPECT, single-photon emission computerized tomography; TR-NIRS, time-resolved near infrared spectroscopy; Xe = xenon.

**TABLE 3:** Summary of articles examining diffuse correlation spectroscopy (DCS) cerebrovascular parameters.

Article	Study subjects	Experimental conditions	Number of subjects	Measure of CBF	NIRS device	Study results and conclusions	Study limitations
**Human studies**							
Durduran et al. (2010)	Human neonates with congenital heart disease	• Group of neonates with congenital heart defects	12	ASL-MRI	Research device (DCS/CW-NIRS hybrid)	• Strong linear Pearson correlation between percent change in CBF_DCS_ and percent change in CBF (R = 0.7; *p* = 0.01)	• CBF was measured globally and not only at region of interest interrogated by DCS.
		• Measurements were taken at various ETCO_2_ levels				• Bland-Altman analysis showed no trend in bias	• Change in CBF only induced through cerebral vasodilation; limits generalizability
							• Measured changes in absorption by CW-NIRS were used to improve CBF_DCS_.
							• Only relative changes in parameters from their baseline were examined
							• Bias assessment incompletely reported
Kim et al. (2010)	Adult brain injury patients	• Group of critically ill adult brain injury patients	7	Xe-CT	Research device	• Strong linear Pearson correlation between percent change in CBF_DCS_ and percent change in CBF (R = 0.73; *p* = 0.010)	• Only relative changes in the parameters from baseline examined
		• Measurements taken during various interventions to modify CBF; included manipulating ABP and ETCO_2_ levels					• No bias assessment performed
Baker et al. (2019)	Adult brain injury patients	• Group of critically ill adult brain injury patients	11	Thermal diffusion flowmetry	Research device (DCS/TR-NIRS hybrid)	• Poor Pearson correlation between CBF_DCS_ and CBF (R = 0.15; no *p*-value reported)	• TR-NIRS measurements required to construct two-layer model used to derive CBF_DCS_.
		• Measurements taken at rest					• Placement of DCS probes not always on same side as thermal diffusion flowmetry probe
							• No bias assessment performed
**Animal studies**							
Zhou et al. (2009)	Newborn piglets	• Porcine model of TBI and a sham injury group	8	Fluorescent microspheres	Research device (DCS/CW-NIRS hybrid)	• Strong linear Pearson correlation between percent change in CBF_DCS_ and percent change in CBF (R = 0.89; *p* < 0.00001)	• Only relative changes parameters from baseline examined
		• Measurements taken at baseline and following injury or sham procedure				• Slope of relationship 1.03 with an intercept of 25.2	• No bias assessment performed
							• CBF measured globally and not only at region of interest interrogated by DCS.
							• CW-NIRS data incorporated to continuously correct for influence of extra axial blood
Carp et al. (2010)	Mature rats	• Group of healthy mature rats	7	ASL-MRI	Research device (DCS/FR-NIRS hybrid)	• Strong linear Pearson correlation between percent change in CBF_DCS_ and percent change CBF (R = 0.86; *p* < 10^−9^)	• Measurement of optical properties of tissue was performed by FR-NIRS to improve accuracy of CBF_DCS_.
		• Measurements taken at various ETCO_2_ levels				• Trend for CBF_DCS_ to underestimate CBF, especially at higher levels	• Change in CBF only induced through cerebral vasodilation; limits generalizability
							• Only relative changes in parameters from baseline examined
							• No bias assessment performed
Shang et al. (2011)	Mature mice	• Mouse model of cerebral ischemia	1	Laser Doppler flowmetry	Research device	• Strong linear Pearson correlation between percent change in CBF_DCS_ and percent change CBF (R = 0.97; *p* < 0.0001)	• Only included a single subject
		• Measurements taken during progressive ischemia				• Slope of relationship 1.40 with intercept of -14.23	• DCS probes we placed directly on skull; limits generalizability in clinical applications
							• No bias assessment performed
Giovannella et al. (2020)	Newborn piglets	• Group of healthy newborn pigs	6	PET imaging	BabyLux (DCS/TR-NIRS hybrid)	• Strong linear Pearson correlation between CBF_DCS_ and CBF (R = 0.94; *p* < 0.0001)	• TR-NIRS measurements required to construct two-layer model used to derive CBF_DCS_.
		• Measurements taken during changes in CBF induced by acetazolamide and during hypoxemia				• Slope of relationship 1.15 with intercept of -1.54	• Measurements between modalities not taken simultaneously
						• Bland-Altman analysis showed minimal bias (0.004) with trend to underestimate CBF by DCS at lower values; 95% limit of agreement ± 0.449	• CBF measured globally and not only at region of interest interrogated by DCS.

ASL-MRI, arterial spin labeling magnetic resonance imaging; CBF_DCS_, cerebral blood flow derived with diffusion correlation spectroscopy; CW-NIRS, continuous-wave near infrared spectroscopy; DCS, diffuse correlation spectroscopy; ETCO_2_ = end-tidal carbon dioxide; PET, positron emission tomography; TR-NIRS, time-resolved near infrared spectroscopy; Xe-CT, xenon-enhanced computed tomography.

The number of subjects in each study varied widely from as few as one (Kirkpatrick et al., 1995; Themelis et al., 2007; Shang et al., 2011) to as many as 60 subjects (Kurth et al., 2002). The majority of studies examined human subjects (Pryds et al., 1990; Skov et al., 1991; Bucher et al., 1993; Kaminogo et al., 1995; Kirkpatrick et al., 1995; Hock et al., 1997; Holzschuh et al., 1997; Villringer et al., 1997; Yamaguchi et al., 1999; Keller et al., 2003; Ogasawara et al., 2003; Rothoerl et al., 2003; Huppert et al., 2006; Murata et al., 2006; Schytz et al., 2009; Durduran et al., 2010; Kim et al., 2010; Taussky et al., 2012; Alderliesten et al., 2014, 2017; Wintermark et al., 2014; Saito et al., 2018; Baker et al., 2019; Milej et al., 2020; Polinder-Bos et al., 2020), and while some were conducted in neonates (Pryds et al., 1990; Skov et al., 1991; Bucher et al., 1993; Durduran et al., 2010; Wintermark et al., 2014; Alderliesten et al., 2017), most were conducted in adults that were either healthy (Villringer et al., 1997; Keller et al., 2003; Huppert et al., 2006; Schytz et al., 2009; Alderliesten et al., 2017; Milej et al., 2020) or in some pathologic state (Kaminogo et al., 1995; Kirkpatrick et al., 1995; Hock et al., 1997; Holzschuh et al., 1997; Yamaguchi et al., 1999; Ogasawara et al., 2003; Rothoerl et al., 2003; Murata et al., 2006; Kim et al., 2010; Taussky et al., 2012; Saito et al., 2018; Baker et al., 2019; Polinder-Bos et al., 2020). Porcine models where the second most popular subjects, with 16 studies utilizing them (Goddard-Finegold et al., 1998; Kuebler et al., 1998; Roberts et al., 1998; Pellicer et al., 1999; Soul et al., 2000; Brown et al., 2001, 2002; Kurth et al., 2002; Ricci et al., 2006; Naulaers et al., 2007; Themelis et al., 2007; Zhou et al., 2009; Elliott et al., 2010, 2014; Alosh et al., 2016; Giovannella et al., 2020). Rodent models were relatively uncommon and were found in only five studies (De Visscher et al., 2003; Pryds et al., 2005; Carp et al., 2010; Shang et al., 2011; Hashem et al., 2022). Only one study was conducted in a canine model (Newton et al., 1997).

There was a wide variety of methods utilized to determine CBF. The majority of animal studies used microsphere (MS) techniques with 7 using radiolabeled MS (Newton et al., 1997; Goddard-Finegold et al., 1998; Kuebler et al., 1998; Roberts et al., 1998; Pellicer et al., 1999; Soul et al., 2000; Ricci et al., 2006), 3 using colored MS (De Visscher et al., 2003; Naulaers et al., 2007; Alosh et al., 2016), and one study using florescent MS (Zhou et al., 2009). This methodology requires sacrifice of the subject and were therefore limited to animal studies. Less invasive radiographic techniques were also commonly used in animal and human subjects including ASL-MRI (Huppert et al., 2006; Carp et al., 2010; Durduran et al., 2010; Wintermark et al., 2014; Alderliesten et al., 2017; Milej et al., 2020; Hashem et al., 2022), PW-MRI (Keller et al., 2003), fMRI-CBF (Alderliesten et al., 2014), CTP (Brown et al., 2001, 2002; Elliott et al., 2010, 2014; Taussky et al., 2012), XeCT (Kim et al., 2010), SPECT (Yamaguchi et al., 1999; Ogasawara et al., 2003; Murata et al., 2006; Schytz et al., 2009), and PET (Hock et al., 1997; Villringer et al., 1997; Saito et al., 2018; Giovannella et al., 2020; Polinder-Bos et al., 2020). Xenon^133^ clearance based methods of determining CBF were also commonly utilized, especially in earlier studies (Pryds et al., 1990; Skov et al., 1991; Bucher et al., 1993; Kaminogo et al., 1995; Holzschuh et al., 1997; Rothoerl et al., 2003). Invasive *in vivo* methods such as laser Doppler flowmetry (Kirkpatrick et al., 1995; Kurth et al., 2002; Pryds et al., 2005; Themelis et al., 2007; Shang et al., 2011) and thermal diffusion flowmetry (Baker et al., 2019) were also utilized.

### Continuous Wave NIRS

As mentioned earlier, of the articles included in this review, CW-NIRS was the most common modality of NIRS utilized. These CW-NIRS studies are summarized in Table 1. This was likely attributable, in part, by the widespread availability of commercially produced CW-NIRS devices. Only 7 of the 28 CW-NIRS studies utilized a device developed in-house (Pryds et al., 1990, 2005; Skov et al., 1991; Goddard-Finegold et al., 1998; Huppert et al., 2006; Kim et al., 2010; Hashem et al., 2022).

The vast majority of studies identified some degree of statistically significant positive Pearson correlation between the CW-NIRS parameter examined and their measure of CBF (R = 0.247 to 0.95; Pryds et al., 1990, 2005; Skov et al., 1991; Bucher et al., 1993; Kaminogo et al., 1995; Kirkpatrick et al., 1995; Hock et al., 1997; Holzschuh et al., 1997; Newton et al., 1997; Villringer et al., 1997; Goddard-Finegold et al., 1998; Yamaguchi et al., 1999; Soul et al., 2000; Ogasawara et al., 2003; Huppert et al., 2006; Murata et al., 2006; Themelis et al., 2007; Taussky et al., 2012; Alderliesten et al., 2014, 2017; Wintermark et al., 2014; Alosh et al., 2016; Polinder-Bos et al., 2020). In those studies that found a significant correlation between CW-NIRS parameters and CBF, the majority were examining a correlation with a relative change in CBF (R = 0.247 to 0.925; Pryds et al., 1990, 2005; Kaminogo et al., 1995; Kirkpatrick et al., 1995; Hock et al., 1997; Holzschuh et al., 1997; Villringer et al., 1997; Yamaguchi et al., 1999; Soul et al., 2000; Ogasawara et al., 2003; Huppert et al., 2006; Themelis et al., 2007; Polinder-Bos et al., 2020), however 10 studies did find a correlation with absolute values of CBF (R = 0.64 to 0.95; Skov et al., 1991; Bucher et al., 1993; Newton et al., 1997; Goddard-Finegold et al., 1998; Murata et al., 2006; Taussky et al., 2012; Alderliesten et al., 2014, 2017; Wintermark et al., 2014; Alosh et al., 2016). The CW-NIRS parameter that was most commonly found to be correlated with either absolute or relative change in CBF was rSO_2_, which was more commonly examined in recent studies (Kaminogo et al., 1995; Yamaguchi et al., 1999; Ogasawara et al., 2003; Taussky et al., 2012; Alderliesten et al., 2014, 2017; Wintermark et al., 2014; Alosh et al., 2016; Polinder-Bos et al., 2020). However, in one of these studies a correlation was only identified in one hemisphere (Polinder-Bos et al., 2020) and in another of these studies a correlation was only present in the sickest subgroup of patients (newborns with severe hypoxic-ischemic encephalopathy; Wintermark et al., 2014). The second most common CW-NIRS parameter was tHgB, which was found to correlate with relative changes in CBF in five studies (Pryds et al., 1990, 2005; Hock et al., 1997; Villringer et al., 1997; Huppert et al., 2006) and absolute CBF in one study (Murata et al., 2006). Notably, in 4 of the earlier studies that identified a correlation with absolute values of CBF, the CW-NIRS parameter was a derived measure of CBF based on the Fick principle, with oxygen utilized as an endogenous tracer (Skov et al., 1991; Bucher et al., 1993; Newton et al., 1997; Goddard-Finegold et al., 1998).

Of note, not all studies found a positive correlation between CW-NIRS parameters and CBF. There were five studies included in this review where either no correlation was identified (Pellicer et al., 1999; Naulaers et al., 2007; Kim et al., 2010; Hashem et al., 2022) or a negative correlation was found between the CW-NIRS parameter and CBF (Ricci et al., 2006; Hashem et al., 2022).

### Dynamic Contrast-Enhanced NIRS

Distinct from other modalities, DCE-NIRS requires the use of an exogenous contrast agent and application of the Fick principle (Gomez et al., 2021c). In all of the articles that leveraged this modality, indocyanine green (ICG) was utilized as the contrast agent (Kuebler et al., 1998; Roberts et al., 1998; Brown et al., 2001, 2002; De Visscher et al., 2003; Keller et al., 2003; Rothoerl et al., 2003; Schytz et al., 2009; Elliott et al., 2010, 2014; Saito et al., 2018; Milej et al., 2020). These studies are summarized in Table 2. Dissimilar to those articles that utilized CW-NIRS, commercially produced devices were used in less than half of the articles (Kuebler et al., 1998; Roberts et al., 1998; Rothoerl et al., 2003; Schytz et al., 2009; Saito et al., 2018).

Most of these studies found a significant positive Pearson correlation between CBF derived from DCE-NIRS (CBF_DCE-NIRS_) and that determined utilizing a “widely accepted” method (Kuebler et al., 1998; Roberts et al., 1998; Brown et al., 2001, 2002; De Visscher et al., 2003; Elliott et al., 2010, 2014; Saito et al., 2018; Milej et al., 2020). In this group CBF_DCE-NIRS_ was correlated with absolute values of CBF (R = 0.6466 to 0.99; Kuebler et al., 1998; Roberts et al., 1998; Brown et al., 2001, 2002; De Visscher et al., 2003; Elliott et al., 2010, 2014; Milej et al., 2020), with the exception of one study which found a correlation between hemispheric ratios of CBF when measured by PET and DCE-NIRS (R = 0.618; *p* = 0.0004; Saito et al., 2018).

There were three studies where a CBF_DCE-NIRS_ was found to not significantly correlate with CBF as measured by PW-MRI (Keller et al., 2003), Xenon^133^ clearance (Rothoerl et al., 2003), or SPECT (Schytz et al., 2009).

### Diffuse Correlation Spectroscopy

The studies included in this scoping review that utilized DCS, a variant of NIRS, to optically probe cerebrovascular physiology are summarized in Table 3. There are a limited number of commercially available DCS devices and as such only a single study was found to use one (Giovannella et al., 2020), while the remaining studies used devices assembled in-house (Zhou et al., 2009; Carp et al., 2010; Durduran et al., 2010; Kim et al., 2010; Shang et al., 2011; Baker et al., 2019). In 5 of the seven studies that derived a CBF utilizing DCS (CBF_DCS_), a strong positive Pearson correlation was found with relative changes in CBF measured by “widely accepted” techniques (R = 0.70 to 0.97; Zhou et al., 2009; Carp et al., 2010; Durduran et al., 2010; Kim et al., 2010; Shang et al., 2011). The most recent study, which was the only one to utilize a commercially available device, was also the only one to find a positive correlation with absolute values of CBF (R = 0.94; *p* < 0.0001; Giovannella et al., 2020). In one study, CBF_DCS_ was not found to be correlated with CBF as measured by thermal diffusion flowmetry (R = 0.15; range -0.35 to 0.60; Baker et al., 2019). Notably, this is the only study in this scoping review that utilized thermal diffusion flowmetry to measure CBF.

### Frequency-Resolved NIRS

There was only one study identified that utilized FR-NIRS. Kurth and others compared CBF, as measured by laser Doppler flowmetry, to rSO_2_, as measured by FR-NIRS, in a cohort of 60 newborn piglets subjected to hyperoxia, hypercapnia, and bilateral carotid occlusion. It was found that rSO_2_, as measured by their in-house FR-NIRS device, had a significant positive correlation with the percent of baseline CBF (R = 0.89; *p* < 0.001). The slope of this relationship was 1.75 with an intercept of -21. Notably, the FR-NIRS probe was placed directly on the skull limiting the generatability of these findings to clinical applications (Kurth et al., 2002).

### Time-Resolved NIRS

There were no studies identified that examined the correlation between a purely TR-NIRS derived parameter and CBF as measured by a “widely accepted” method.

## Discussion

Through this systematic scoping review of the literature, 47 studies have been identified that quantitatively examine the relationship between cerebrovascular parameters, as measured by NIRS, and CBF, as measured by “widely accepted” methods. The most popular modality studied in the literature was CW-NIRS, likely attributable to the number of commercially available devices and ease of use (Pryds et al., 1990, 2005; Skov et al., 1991; Bucher et al., 1993; Kaminogo et al., 1995; Kirkpatrick et al., 1995; Hock et al., 1997; Holzschuh et al., 1997; Newton et al., 1997; Villringer et al., 1997; Goddard-Finegold et al., 1998; Pellicer et al., 1999; Yamaguchi et al., 1999; Soul et al., 2000; Ogasawara et al., 2003; Huppert et al., 2006; Murata et al., 2006; Ricci et al., 2006; Naulaers et al., 2007; Themelis et al., 2007; Kim et al., 2010; Taussky et al., 2012; Alderliesten et al., 2014, 2017; Wintermark et al., 2014; Alosh et al., 2016; Polinder-Bos et al., 2020; Hashem et al., 2022). DCE-NIRS (Kuebler et al., 1998; Roberts et al., 1998; Brown et al., 2001, 2002; De Visscher et al., 2003; Keller et al., 2003; Rothoerl et al., 2003; Schytz et al., 2009; Elliott et al., 2010, 2014; Saito et al., 2018; Milej et al., 2020) and DCS (Zhou et al., 2009; Carp et al., 2010; Durduran et al., 2010; Kim et al., 2010; Shang et al., 2011; Baker et al., 2019; Giovannella et al., 2020) methodologies were commonly reported on as well. This is despite their more complicated protocols and setups. There was only one study that examined the relationship between FR-NIRS and measures of CBF (Kurth et al., 2002), while no study compared TR-NIRS parameters directly with CBF. The relative advantages and limitations of each NIRS modality that were identified through this review have been summarized in Table 4.

**TABLE 4:** Summary of limitations and advantages of various NIRS modalities identified through this review.

NIRS modality	Advantages	Limitations
CW-NIRS	• Robust literature supporting a positive linear correlation between change in derived parameters and change in CBF	• Limited literature supporting association with absolute values of CBF
	• Standardized parameters such at tHgB and rSO_2_ are reproducible associated with changes CBF	• Many studies that identifying an association with absolute values of CBF commonly utilized oxygen tracer
	• Most studies based on commercially available devices	
DCE-NIRS	• Majority of literature supports an association with absolute values of CBF	• Most studies utilized devices developed in house
		• Limited temporal resolution due to the need of an exogenous contrast agent
DCS	• Literature supporting a positive linear correlation between change in derived parameters and change in CBF	• All but one study utilized devices developed in house
		• One identified an association with absolute values of CBF
FR-NIRS	• The single study identified had a large cohort and supported an association between derived parameters and change in CBF	• Single study identified through systematic review
	• Significant potential for further exploration given the limited body of literature	
TR-NIRS	• Significant potential for further exploration given the limited body of literature	• No studies identified through systematic review

CBF, cerebral blood flow; CW-NIRS, continuous wave near infrared spectroscopy; DCE-NIRS, dynamic contrast-enhanced near infrared spectroscopy; DCS, diffuse correlation spectroscopy; FR-NIRS, frequency-resolved NIRS; NIRS, near infrared spectroscopy; rSO_2_ = regional cerebral oxygen saturation; tHgB = total hemoglobin; TR-NIRS, time-resolved near infrared spectroscopy.

Most of the studies identified examined human subject (Pryds et al., 1990; Skov et al., 1991; Bucher et al., 1993; Kaminogo et al., 1995; Kirkpatrick et al., 1995; Hock et al., 1997; Holzschuh et al., 1997; Villringer et al., 1997; Yamaguchi et al., 1999; Keller et al., 2003; Ogasawara et al., 2003; Rothoerl et al., 2003; Huppert et al., 2006; Murata et al., 2006; Schytz et al., 2009; Durduran et al., 2010; Kim et al., 2010; Taussky et al., 2012; Alderliesten et al., 2014, 2017; Wintermark et al., 2014; Saito et al., 2018; Baker et al., 2019; Milej et al., 2020; Polinder-Bos et al., 2020). Neonates were examined in a minority of studies which is surprising, as their thin scalp and skull are thought to render the neonatal brain particularly well suited for examination with NIRS (Dix et al., 2017). This may be a product of the strict inclusion criteria of this scoping review, that required comparison with a “widely accepted” measure of CBF, a practically challenging feat in neonates, especially those that are premature (Pryds and Edwards, 1996). Animal models were also commonly used with porcine models being the most popular in the studies included in this review (Goddard-Finegold et al., 1998; Kuebler et al., 1998; Roberts et al., 1998; Pellicer et al., 1999; Soul et al., 2000; Brown et al., 2001, 2002; Kurth et al., 2002; Ricci et al., 2006; Naulaers et al., 2007; Themelis et al., 2007; Zhou et al., 2009; Elliott et al., 2010, 2014; Alosh et al., 2016; Giovannella et al., 2020). Given that large animal models provide a closer approximation to human brains due to their similar cerebrovascular structure and gyrencephalic nature the presence of very few small animal studies can be a seen as a relative strength of this body of literature (Sorby-Adams et al., 2018). The requirement of most NIRS devices to have an emitter and detector that are separated in space may have contributed to this preference for human and large animal models. Generally, subject numbers were small with the largest study having 60 participants (Kurth et al., 2002). Once again, this is likely due to the significant resource intensive nature of any study comparing NIRS cerebrovascular parameters to a “widely accepted” measure of CBF.

A variety of “widely accepted” methods of determining true CBF were utilized in the studies included in this scoping review. Despite the heterogeneity, most methodologies either had a defined region over which CBF was being measured or the ability to define a region of interest. This is of particular importance when comparing these values to NIRS parameters as NIRS technology, by its nature, evaluates regional cerebrovascular physiology and is not a global measure. That said, not all studies aimed to match these regions of interest when evaluating associations, and as such, these studies tended to fail in identifying a relationship between parameters (Pryds et al., 1990; Skov et al., 1991; Bucher et al., 1993; Kaminogo et al., 1995; Holzschuh et al., 1997; Goddard-Finegold et al., 1998; Pellicer et al., 1999; De Visscher et al., 2003; Ricci et al., 2006; Naulaers et al., 2007; Zhou et al., 2009; Durduran et al., 2010; Giovannella et al., 2020).

### Continuous Wave NIRS

There appears to be relatively robust literature supporting some degree of positive linear association between CW-NIRS parameters and CBF. Some of the most compelling associations, especially when only considering absolute values of CBF, were found when the Fick principle was utilized with oxygen as a tracer (Skov et al., 1991; Bucher et al., 1993; Newton et al., 1997; Goddard-Finegold et al., 1998). This method requires a sudden increase in oxygen saturation, and as such, can only be utilized as an intermittent method to evaluate CBF. Additionally, these studies assume a constant rate of oxygen consumption and ignored any possible effect a sharp increase in oxygenation may have on cerebral vasculature.

Several CW-NIRS parameter were examined for their association with CBF in the identified literature. This included OxHgB (the concentration of oxygenated hemoglobin in the tissue interrogated), deOxHgB (the concentration of deoxygenated hemoglobin in the tissue interrogated), tHgB (the total concentration of hemoglobin in the tissue interrogated), as well as TOI and RSO_2_ (which are both ratios of the concentrations of oxygenated hemoglobin to total hemoglobin in the tissue interrogated). Additionally, ΔHgB (the difference between the concentrations of OxHgB and deOxHgB in the tissue interrogated) was also examined.

The parameter of tHgB seemed to mostly be associated with changes in CBF and not absolute values of CBF (Pryds et al., 1990, 2005; Hock et al., 1997; Villringer et al., 1997; Huppert et al., 2006). This may not be entirely surprising as changes in the totally concentration of hemoglobin in a volume of brain would be expected to increase with increased CBV. Somewhat surprisingly, rSO_2_ was frequently found to have a linear relationship with CBF, especially when also considering changes from some baseline (Kaminogo et al., 1995; Holzschuh et al., 1997; Yamaguchi et al., 1999; Ogasawara et al., 2003; Taussky et al., 2012; Alderliesten et al., 2014, 2017; Wintermark et al., 2014; Alosh et al., 2016; Polinder-Bos et al., 2020). An interesting finding of this study is that this relationship seems to breakdown in hypothermic subjects (Naulaers et al., 2007; Wintermark et al., 2014). However, this was only found in two studies and will therefore need further validation. Given that rSO_2_ is the ratio of OxHgB to tHgB this may indicate that changes in CBF are primarily driven by arteriolar dilation or contraction and have a linear effect on the ratio of OxHgB to tHgB, over some range. The disproportionate change in OxHgB compared to deOxHgB with changes in CBF is further evidenced by two studies that found that changes ΔHgB correlated well with changes in CBF, once again indicating that changes in CBF my primarily be secondary to increases in arteriolar volume (Soul et al., 2000; Pryds et al., 2005).

A total of five studies failed to find any association between CBF and CW-NIRS parameters (Pellicer et al., 1999; Ricci et al., 2006; Naulaers et al., 2007; Kim et al., 2010; Hashem et al., 2022). It is important to note that in three of these studies CW-NIRS parameters were compared to global CBF as opposed to in the region of interest examined by NIRS (Pellicer et al., 1999; Ricci et al., 2006; Naulaers et al., 2007). In the study by Hashem and others, atypical methods were utilized to derive the CW-NIRS parameters that involved the assumption that tHgB was equal to deOxHgB during an induced anoxic pulse (Hashem et al., 2022). Finally, in the study by Kim and others, sample size was small, and a moderate correlation was found but failed to meet statistical significance (Kim et al., 2010).

### Dynamic Contrast-Enhanced NIRS

In studies that utilized DCE-NIRS, the contrast agent of choice was ICG. Generally, there was a strong association between absolute CBF_DCE-NIRS_ and CBF as measured by “widely accepted” methods. However, there were exceptions to this. In the study conducted by Keller and others, the limits of agreement were unacceptably large, but this is likely attributable to a small sample size and an inability to carry out measurements simultaneously with both modalities (Keller et al., 2003). In the article by Rothoerl and others, rSO_2_ was used to monitor the progression of the ICG contrast (Rothoerl et al., 2003). This is particularly unusual since rSO_2_ is a function of the ratio of OxHgB to tHgB, both of which would be affected by a change in optical density caused by ICG. Finally, it is likely that methodologic errors were at play in the article by Schytz and others as there was a failure to detect any change in CBF_DCE-NIRS_ before and after the administration of acetazolamide (Schytz et al., 2009).

There are limitations to this methodology. As with any measure of CBF utilizing the Fick principle, DCE-NIRS is limited to only intermittent measurements of CBF. While the temporal resolution of these methods is likely limited by contrast washout rates, practically, frequent measurements may primarily be limited by the need for a contrast agent altogether. Additionally, two of the studies required radiographic imaging to determine the thickness of extracerebral tissue prior to the measurement of cerebrovascular parameters (Elliott et al., 2010; Milej et al., 2020).

### Diffuse Correlation Spectroscopy

Measures of change in CBF generally correlated well with changes in CBF_DCS_ however, there were few studies that examined this relationship. The one study, by Baker and others, that failed to find any association was limited by the fact that DCS probes were not always placed on the same side as the thermal diffusion flowmetry probes (Baker et al., 2019). Additional validation of these findings will be required before any definitive conclusions can be drawn.

### Frequency-Resolved NIRS

In the study by Kurth and others, a strong association was found between rSO_2_ and change in CBF from baseline. While this study contained the largest number of subjects of any study in this review, it is still only a single study. Additionally, in this study the optical probe was placed directly on the skull, limiting its generalizability to the clinical setting (Kurth et al., 2002).

It should also be noted that in the DCS study by Carp and others, FR-NIRS was utilized to determine the optical properties of the tissues and improve the measurement of CBF_DCS_ (Carp et al., 2010).

### Time-Resolved NIRS

While no studies examined the association between TR-NIRS parameters and CBF, the technology was utilized in two studies to improve the accuracy of CBF_DCS_ measurements (Baker et al., 2019; Giovannella et al., 2020). Additionally, two studies using DCE-NIRS utilized TR-NIRS to measure changes caused by the contrast bolus (Elliott et al., 2014; Milej et al., 2020).

### Limitations of the Literature

When considering limitations of this body of literature it is important to consider the statistical methodology utilized to evaluate the association between CBF and NIRS cerebrovascular parameters. Most studies assessed for simple correlations between parameters. In some cases, only the correlation between changes from baseline were evaluated. While this does provide evidence that NIRS parameters of cerebrovascular physiology move with CBF, any correlation analysis does not provide information about potential confounders.

One obvious confounder is interference due to extracerebral tissue. If a change in CBF is induced that has a concurrent change in scalp blood flow a positive correlation might be seen even if the NIRS signal is primarily influenced by the scalp. While some studies utilized hybrid technologies or spatially resolved techniques to handle interference from the scalp others simply removed the scalp in their animal model. Of note, one study was able to examine this relationship and found that signals from the scalp did not correlate with NIRS signals in their experimental setup (Kuebler et al., 1998).

Another source of error can be the various experimental conditions that effect CBF along with another cerebrovascular parameter that are more directly detected by NIRS. An example of this would be hypercapnic induced elevations in CBF. This experimental condition was utilized in numerous experiments and is accompanied by cerebral vasodilation, thereby increasing cerebral blood volume (CBV). It is unclear, in these experiments, if it is perhaps a change in CBV that is being detected, especially in the setting of CW-NIRS devices. The vasoactive effects of oxygen and xenon, similarly, have not been accounted for in a number of studies (Skov et al., 1991; Bucher et al., 1993; Newton et al., 1997; Goddard-Finegold et al., 1998; Kim et al., 2010). This is important to consider as it may not be the mechanism by which all increases, or decreases, in CBF occur.

CBF has a complex regulatory mechanism that can be influenced by numerous factors including neuronal activity, systemic blood pressure, pharmacologic agents, and various pathologic states (Slupe and Kirsch, 2018; Liu et al., 2020a; Korte et al., 2020). CBF can also be measured on the scale of large arteries or on the scale of the microvasculature. Given that NIRS primary examines CBF at the microvascular level, concordance with method of measuring CBF at the arterial level maybe limited, especially considering that the factors that influence CBF on the arterial scale (Leng et al., 2017; Liu et al., 2018) can be distinct from those that influence microvascular CBF (Tikhomirova et al., 2011; Liu et al., 2021).This difference in scale of measurement has not been fully explored in the literature despite NIRS becoming increasingly substituted as an alternative method of evaluating CBF in clinical studies(Yang et al., 2019; Tian et al., 2021; Guo and Chen, 2022).

The articles identified by this review generally failed to perform comparative analysis beyond a simple correlation. While some did perform a Bland-Altman analysis, it was often incomplete and did not assess a trend in the bias. Additionally, in those situations where CBF could be measured continuously, such as with laser Doppler flowmetry, alongside NIRS parameters, there was no attempt at time series analysis.

Finally, a major limitation to the articles identified by this systematically conducted scoping review is the generally small cohort sizes. As mentioned previously, this is likely due to the cumbersome nature of the methods involved. It is difficult to have confidence in conclusions drawn from the studies identified in this review with so few data points. It should be acknowledged that a wider body of literature that has examined the use of NIRS based methods to measure blood flow in other tissues does exist and should, to some degree, bolster confidence in the findings of the studies identified in this review (Choo et al., 2017; Bangalore-Yogananda et al., 2018; Dennis et al., 2021).

### Limitations of This Review

This systematically conducted review aimed to provide a scoping overview of the literature examining the relationship between CBF and various NIRS parameters. There are, however, inherent limitations to this review. Given the heterogeneity of the experimental designs, subjects, and modalities utilized a meta-analysis of the data was not possible. Even within similar NIRS modalities, studies were significantly different in their experimental conditions such that a meta-analysis would not be feasible or appropriate. Additionally, the decision of what methods of measuring CBF were acceptable was somewhat arbitrary. An argument could be made to include only those studies that utilized rigorous microsphere techniques and Xenon^133^ clearance. Conversely, it could also be argued that methods such as TCD and BOLD-fMRI provide reasonable enough methods of measuring CBF despite obvious confounders. This study aimed to strike a balance and include methods that had reasonably accurate measurements of CBF and not just a related cerebrovascular physiologic parameter. One corollary of the methods that were determined to be “widely acceptable” measures of CBF is that there are inconsistencies between what each modality is measuring. Of note, laser Doppler flowmetry is more accurately a measure of CBF velocity at the cortical surface and as such, does not entirely measure the volume of blood passing through a mass of brain tissue. The result is that certain interventions that are aimed to modulate CBF, such as administration of vasodilators, may increase CBF while reducing CBF velocity. Further to this point, certain “widely-accepted” methods of measuring CBF, such as CTP and ASL-MRI, primarily measure CBF in large cerebral vessels. This contrasts with NIRS modalities that primarily in the cerebral microvasculature (arterioles, capillaries, and venules). As a result, concordance between these methods should not entirely be strictly expected as various interventions and pathologic states may affect these differently.

Finally, given the overall globalization of scientific research, the inclusion of only English-language studies limits the available literature for inclusion in this review. This was simply made as a pragmatic decision based on the limitations of the authors involved in this study.

### Future Directions

The results of this scoping review of the literature provide some guidance for future avenues of exploration. Given the large body of evidence supporting the association between CW-NIRS parameters and CBF, there should be a degree of confidence in research that utilizes these modalities as a means of interrogating changes in CBF as a component of cerebral multimodal monitoring. Such clinical research is already ongoing (Kim et al., 2014; Weigl et al., 2014; Trofimov et al., 2016). Beyond this, CW-NIRS parameters likely serve as an ideal non-invasive method of continuously monitoring cerebral autoregulation. These methods are continuously updating indices that examine the correlation between arterial blood pressure (ABP) and CW-NIRS parameters (Zeiler et al., 2017). These indices are a particularly well-suited application of CW-NIRS as they, by design, rely only on the relative change in CBF and not absolute values of it. They have been shown in animal models to accurately determine the lower limit of cerebral autoregulation (Brady et al., 2007, 2010). This is now being used as a method of not only measuring cerebral autoregulation in the acute setting but, due to its non-invasive nature, is being explored as a means of tracking the recovery of cerebral autoregulation in the chronic phase (Gomez et al., 2020b; 2020a).

Accurate evaluations of absolute CBF are likely to be derived from modalities of NIRS that leverage the Fick principle. This may be done through CW-NIRS methods in which oxygen is utilized as an endogenous tracer or DCE-NIRS where ICG is used as an exogenous contrast agent. These methods, as mentioned previously, are limited by their temporal resolution but may be more suitable for applications, both research and clinical, that require only “moment in time” measurement of CBF and therefore may be a viable alternative to cumbersome, low temporal resolution methods such as Xe-CT, ASL-MRI or PET imaging.

The literature around DCS, FR-NIRS, and TR-NIRS is relatively scant with regards to their ability to accurately measure CBF, or correlate with CBF changes. Prior to these modalities being accepted as non-invasive measures of CBF more work is needed. Studies involving large cohorts that utilize more sophisticated methods of comparison with “widely accepted” measures of CBF need to be conducted to validate the body of literature currently supporting their use.

Finally, given that the regulation of CBF on both the arterial and microvascular scale is complex, with numerous variables affecting flow, non-linear computational models that describe cerebral hemodynamics in various conditions may provide vital insights into the precise relationship between CBF and NIRS based metrics (Liu et al., 2020b; Xie et al., 2022). Examining NIRS based metrics in light of these computational models deserves further exploration.

## Conclusion

In this scoping review of the literature examining the quantitative relationship between CBF and NIRS- based cerebrovascular measures, CW-NIRS was the most examined modality with DCE-NIRS, DCS, and FR-NIRS also being examined, but to a lesser degree. The identified studies almost exclusively examined this relationship with correlation analysis. There was a significant amount of literature supporting a positive linear relationship between changes in CW-NIRS based metrics, particularly rSO_2_, and changes in CBF. Linear relationships were also identified between other NIRS based modalities and CBF, however, further validation is needed.

## Data Availability

The original contributions presented in the study are included in the article/Supplementary Material, further inquiries can be directed to the corresponding author.
