# Supplementary material for: The Quantitative Associations Between Near Infrared Spectroscopic Cerebrovascular Metrics and Cerebral Blood Flow: A Scoping Review of the Human and Animal Literature
Source: Front Physiol. 2022 Jul 15;13:934731. doi: 10.3389/fphys.2022.934731 (PMC9335366; doi:10.3389/fphys.2022.934731)
Supplement: Supplementary file 1 [file DataSheet1.DOCX]

# Supplementary Material

**Appendix A: Search Strategy for BIOSIS**

**TS = (“Near infrared Spectroscopy” OR “Near-Infrared Spectroscopies” OR “Near-Infrared Spectroscopy” OR “NIRS” OR “Spectroscopies, Near-Infrared” OR “Spectroscopy, Near Infrared” OR “NIR Spectroscopy” OR “NIR Spectroscopies” OR “Spectroscopies, NIR” OR “Spectroscopies, NIR” OR “Spectrometry, Near-Infrared” OR “Near-Infrared Spectrometries” OR “Near-Infrared Spectrometry” OR “Spectrometries, Near-Infrared” OR “Spectrometry, Near Infrared” OR “Deoxyhemoglobin” OR “Deoxygenated Hemoglobin” OR “Oxyhemoglobin” OR “Oxygenated Hemoglobin” OR “Total Hemoglobin” OR “Regional oxygen saturation” OR “Cerebral Oxygen Saturation” OR “Cerebral Oxygenation” OR “RSO2” OR “Tissue Oxygen Index” OR “TOI” OR “Total Hemoglobin Index” OR “THI” OR “Brain Oxygen Saturation” OR “Diffuse correlation spectroscopy” OR “DCS”)**

**AND**

**TS = (Cerebral Blood Flow OR CBF OR Blood flow, cerebral OR Brain blood flow OR Regional cerebral blood flow OR Regional CBF OR Global Cerebral Blood flow OR Global CBF OR Cerebral Circulation OR Cerebral perfusion OR Positron Emission Tomography OR PET OR Single-photon emission computer tomography OR Single photon emission computer tomography OR Single-photon emission CT OR Single photon emission CT OR SPECT OR Xenon computer tomography OR Xenon CT OR 131Xe computer tomography OR 131Xe CT OR Laser Doppler Flowmetry OR LDF OR Perfusion computer tomography OR computer tomography, perfusion OR Perfusion CT OR CT Perfusion OR Magnetic resonance perfusion OR MR perfusion OR Perfusion magnetic resonance OR Perfusion MR)**
